# Dynamics of *Streptococcus pneumoniae* Serotypes Causing Acute Otitis Media Isolated from Children with Spontaneous Middle-Ear Drainage over a 12-Year Period (1999–2010) in a Region of Northern Spain

**DOI:** 10.1371/journal.pone.0054333

**Published:** 2013-01-22

**Authors:** Marta Alonso, José M. Marimon, María Ercibengoa, Eduardo G. Pérez-Yarza, Emilio Pérez-Trallero

**Affiliations:** 1 Microbiology Department, Hospital Universitario Donostia-Instituto Biodonostia, San Sebastián, Spain; 2 Biomedical Research Center Network for Respiratory Diseases, San Sebastián, Spain; 3 Pediatric Department, Hospital Universitario Donostia-Instituto Biodonostia, San Sebastián, Spain; 4 Faculty of Medicine, University of the Basque Country, San Sebastián, Spain; Rockefeller University, United States of America

## Abstract

The aim of this study was to determine the serotype and clonal distribution of pneumococci causing acute otitis media (AOM) and their relationship with recurrences and mixed infections with other microorganisms under the influence of the 7-valent pneumococcal conjugate vaccine (PCV7). To do this, all pneumococcal isolates collected from the spontaneous middle-ear drainage of children <5 years old diagnosed of AOM by their pediatrician or their general practitioner from 1999 to 2010 were phenotypically characterized and the most frequent serotypes were genotyped. In the 12-year study, 818 episodes of pneumococcal AOM were detected, mostly (70.5%) in children younger than 2 years old. In 262 episodes (32%), the pneumococci were isolated with another bacterium, mainly (n = 214) *Haemophilus influenzae*. Mixed infections were similar in children under or over 2 years old. The most frequent serotypes were 19A (n = 227, 27.8%), 3 (n = 92, 11.2%) and 19F (n = 74, 9%). Serotypes included in the PCV7 sharply decreased from 62.4% in the pre-vaccination (1999–2001) to 2.2% in the late post-vaccination period (2008–2010). Serotype diversity steadily increased after the introduction of the PCV7 but decreased from 2008–2010 due to the predominant role of serotype 19A isolates, mostly ST276 and ST320. The prevalence of serotype 3 doubled from 6.1% (20/326) in 1999–2004 to 14.6% (72/492) in 2005–2010. Relapses mainly occurred in male infants infected with isolates with diminished antimicrobial susceptibility. Reinfections caused by isolates with the same serotype but different genotype were frequent, highlighting the need for genetic studies to differentiate among similar strains. In conclusion, the main change in pneumococcal AOM observed after the introduction of the PCV7 was the sharp decrease in vaccine serotypes. Also notable was the high burden of serotype 19A in total pneumococcal AOM before and especially after the introduction of the PCV7, as well as in relapses and reinfections.

## Introduction

Acute otitis media (AOM) is one of the most common infections during childhood and nearly every child has experienced an episode of AOM by the age of 5 years [1], [2]. The etiology of AOM varies with age, the most frequently implicated agents being viruses such as rhinoviruses, influenza viruses, or respiratory syncytial viruses and bacteria, such as non-encapsulated *Haemophilus influenzae*, *Streptococcus pneumoniae* and *Moraxella catarrhalis*. Due to the high frequency of AOM, this infection accounts for one of the highest expenditures in health care, including direct (physician visits and antibiotics) or indirect (lost hours of work, etc.) costs [3]. *S. pneumoniae* is one of the main agents causing bacterial AOM, directly or as complication of a viral upper respiratory tract infection [4]. Since the introduction of the 7-valent pneumococcal conjugate vaccine (PCV7) for the prevention of invasive pneumococcal disease, many researchers have demonstrated a reduction in AOM cases in vaccinated as well as in non-vaccinated children as a consequence of herd protection [2], [5]. However, an increase in the proportion of AOM caused by non-vaccine serotypes, especially of serotype 19A, has also been reported [5], [6].

The main aim of this work was to analyze the epidemiology of pneumococcal AOM infection in relation to the serotype and clonal distribution of isolates in San Sebastián, northern Spain, over a 12-year period. As a secondary aim, the frequency of coinfection with other bacteria and the possible association of specific pneumococcal serotypes with recurrences were also studied.

## Methods

### Patients and Isolates

The study was conducted at Hospital Universitario Donostia, located in the city of San Sebastián, Basque Country, northern Spain which attends an estimated population of around 350,000 inhabitants from the region of Donostialdea and adjacent areas. The study included all *S. pneumoniae* isolates recovered from 1999 to 2010 from the spontaneous middle-ear drainage of children aged <5 years old diagnosed of AOM by their pediatrician or their general practitioner. Samples were sent to the Microbiology Department of Donostia Hospital where all microbiological procedures were performed. Publication of the results was approved by the Ethical Committee for Clinical Research of the Health Area of Gipuzkoa with a waiver of informed consent documentation since this was a retrospective study and patients’ identities were safeguarded.

The middle ear fluid was processed by Gram-staining and culture. Pneumococci were identified by their colony morphology, optochin susceptibility and bile solubility. Bacteria others than *S. pneumoniae* were identified using standard microbiological procedures (requirement of X and V factors for growth of *H. influenzae* and coaglutination with specific b and a, c-f antisera; bacitracin-susceptibility and latex agglutination with specific group A streptococci antisera for *Streptococcus pyogenes;* oxidase-test and carbohydrate fermentation tests for *M. catarrhalis*; coagulase test for *Staphylococcus aureus*). Serotyping was performed with the Quellung reaction, an antibody-based microarray serotyping technique (Pneumoarray, Abyntek, Spain) and/or multiplex polymerase chain reaction (PCR) [7]. Isolates giving a negative serotype reaction and not amplifying the capsular-gene locus [8] were defined as non-encapsulated. The serotype of all 19F and 19A isolates serotyped with the Quellung reaction or the Pneumoarray was confirmed by a PCR specific for the 19F and 19A capsular loci [9].

This study was performed between 1999 and 2010 and was divided into four time periods: one pre-vaccination period, from 1999 to 2001 and three post-PCV7 periods: from 2002 to 2004 (early post-PCV7 period), from 2005 to 2007 (intermediate post-PCV7 period) and from 2008 to 2010 (late post-PCV7 period). The PCV7 has not been included in the official vaccination schedule of the public health system of the Basque Country, and was only dispensed in private practice. Its use had progressively increased from 2002, with a vaccine coverage rate in children under 2 years of age estimated to be close to 50% before 2006 [10]) and 60% in 2009, based on the number of doses sold in Gipuzkoa.

Minimal inhibitory concentrations were determined by the broth microdilution assay according to Clinical and Laboratory Standards Institute (CLSI) guidelines [11].

### Molecular Characterization of Pneumococcal Isolates

To study the clonal relationship of the most frequent serotypes, pulsed-field gel electrophoresis (PFGE) and multi-locus sequence typing (MLST) were performed. PFGE was carried out according to previously described protocols [12] and MLST according to the protocol described on the pneumococcal MLST web site (http://www.mlst.net). PFGE characterization was performed in a random sample of approximately half of all isolates of the two most prevalent serotypes. Moreover, PFGE was also performed in all isolates causing relapses or reinfections by the same serotype in the same children and in selected multidrug-resistant isolates (penicillin MIC >0.06 µg/ml and non-susceptibility to a further two or more antimicrobial classes). PFGE similarity patterns of <85% were considered as different and were arbitrarily named in this study with a capital letter, from A to Z. After *Sma*I digestion, PFGE patterns were analyzed and a dendrogram was constructed using the Diversity Database software (Bio-Rad Laboratories, USA), with a band tolerance of 1%, the Dice coefficient and the unweighted pair group method with arithmetic averages (UPGMA). Isolates of the most prevalent PFGE patterns were further characterized by determining the MLST of two to three representative isolates.

### Statistical Analyses

Differences in the distribution of serotypes were analyzed by the chi squared test or by Fisher’s exact test when appropriate. Analysis of trends over time was calculated using the chi squared test for trend (GraphPad Instat ver 3.05. La Jolla, CA, USA). A p value of <0.05 was considered as statically significant. Diversity was calculated by Simpson’s Index of Diversity 1**–** D, where D = ∑n * (n−1)/N * (N−1), with n being the number of isolates of a specific serotype in each period and N the total number of isolates (sample size) in each period. The on-line tool: darwin.phyloviz.net/ComparingPartitions/was used. The value of this index ranges between 0 and 1 and the greater the value, the greater the sample diversity.

### Recurrences, Relapses and Reinfections

In this study, recurrences encompassed both relapses and reinfections: a relapse was defined as a second episode of AOM caused by the same serotype and genotype with an interval between 7 and 89 days from the first episode. A reinfection was defined as a second episode produced by a different serotype or by the same serotype but with a different genotype, or as a second episode caused by the same serotype and genotype but taking place after 90 or more days. In this study, reinfections, but not relapses, were included as distinct episodes.

## Results

Between 1999 and 2010, 1,006 non-duplicated *S. pneumoniae* isolates were cultured from the middle ear exudates collected from patients diagnosed with AOM. Of these, 818 (81.3%) belonged to 708 children aged <5 years old. A single episode of pneumococcal AOM was observed in 622/708 (87.9%) children and two or more episodes in 86 (12.1%) (Table 1). Age at the time of the first AOM episode, was less than 1 year in 229 infants (55.9% boys), 1 year in 270 (54.1% boys) children, 2 years in 105 (56.2% boys), 3 years in 63 (58.7% boys) children and 4 years in 41 (63.4% boys) children. By episodes, 69.1% (565/818) occurred before the child’s second birthday.

**Table 1:** General characteristics of patients and pneumococcal acute otitis media (AOM) episodes.

No. patients (% males)	708 (55.9)
No. episodes	818
Age of patients in months at the time of the first AOM episode [median (interquartile range)]	12 (10–24)
No. (%) episodes with *Streptococcus pneumoniae* alone	556 (68.0)
No. (%) episodes with mixed infections	262 (32.0)
No. (%) patients with only 1 AOM episode	622 (87.8)
No. patients with reinfections	86
No. patients with 1 episode of reinfection	68
No. patients with 2 episodes of reinfection	13
No. patients with 3 episodes of reinfection	4
No. patients with 5 episodes of reinfection	1
*No. patients with relapses* 1	*21*
*No. patients relapsing with an interval of 7–20 days*	*14*
*No. patients relapsing with an interval of 21–30 days*	*6*
*No. patients relapsing with an interval of 31–89 days*	*1*

1 Relapses were not included as distinct episodes.

### Serotype Distribution

Overall, the three most frequent serotypes were 19A (n = 227, 27.8%), 3 (n = 92, 11.2%) and 19F (n = 74, 9.0%).

**Figure 1:**
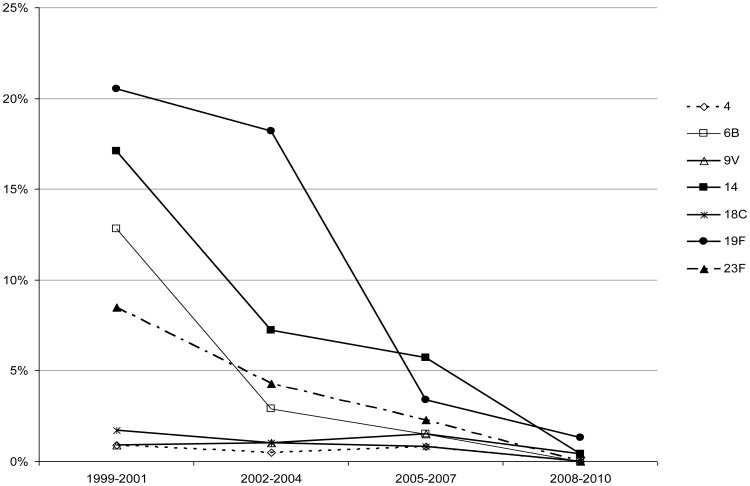
Percentage evolution of serotypes (number of a specific serotype/total isolates in each period) included in the 7-valent pneumococcal conjugate vaccine.

The prevalence of PCV7 serotypes causing AOM sharply decreased after the introduction of the PCV7 in 2001, from 62.4% in the 1999–2001 pre-vaccination period to 2.2% in the 2008–2010 late post-vaccination period (p value for trend p<0.001). Individually, each of the four most prevalent PCV7 serotypes (6B, 14, 19F and 23F) significantly decreased from 1999–2001 to 2008–2010 (p<0.001), with the fall in the percentage of serotypes 19F, 14, 6B and 23F across the four study periods being particularly striking (Fig. 1). The prevalence of serotypes 1, 5 and 7F, included in the 10-valent PCV (PCV10) but not in the PCV7, was low (equal to or under 4% for each serotype); however, when considered all together, these serotypes showed an increasing trend over the study period (p for trend p = 0.003). The number of AOM caused by the two most prevalent serotypes (3 and 19A), included in the PCV13 but not in the PCV7 nor in the PCV10, also increased throughout the study period (p for trend p<0.001 for each serotype). Specifically, the prevalence of serotype 19A increased from 17.9% to 37.9% between 1999–2001and 2008–2010 (p<0.001) and that of serotype 3 increased from 5.1% to 15.0% (p = 0.007). AOM caused by serotype 6A did not vary but those caused by serotype 6C showed a striking increase (p for trend p<0.001). Only 9 of the 818 pneumococci were non-encapsulated.

### Serotype Diversity

The overall number of different serotypes causing pneumococcal AOM in young children increased after the introduction of the PCV7 (Table 2). In the 1999–2001 period, only 21 serotypes were identified but in the next three periods, 31 or more serotypes were found (33 in 2002–2004, 38 in 2005–2007 and 31 in 2008–2010).

**Table 2:** Serotype distribution of pneumococci isolated from 818 episodes of acute otitis media from children less than 5 years of age in Gipuzkoa, Northern Spain (1999–2010).

Serotype	1999–2001		2002–2004		2005–2007		2008–2010		Total	
	n	%	n	%	n	%	n	%	n	%
4	1	0.9	1	0.5	2	0.8	0		4	0.5
6B	15	12.8	6	2.9	4	1.5	0		25	3.1
9V	1	0.9	2	1.0	4	1.5	1	0.4	8	1.0
14	20	17.1	15	7.2	15	5.7	1	0.4	51	6.2
18C	2	1.7	2	1.0	2	0.8	0		6	0.7
19F	24	20.5	38	18.2	9	3.4	3	1.3	74	9.0
23F	10	8.5	9	4.3	6	2.3	0		25	3.1
**PCV7** 1	**73**	**62.4**	**73**	**34.9**	**42**	**15.8**	**5**	**2.2**	**193**	**23.6**
1	1	0.9	3	1.4	10	3.8	7	3.1	21	2.6
5	0		0		2	0.8	5	2.2	7	0.9
7F	1	0.9	7	3.3	8	3.0	9	4.0	25	3.1
**PCV10** 2	**2**	**1.7**	**10**	**4.8**	**20**	**7.5**	**21**	**9.3**	**53**	**6.5**
3	6	5.1	14	6.7	38	14.3	34	15.0	92	11.2
6A	2	1.7	8	3.8	9	3.4	4	1.8	23	2.8
19A	21	17.9	53	25.4	67	25.3	86	37.9	227	27.8
**PCV13** 3	**29**	**24.8**	**75**	**35.9**	**114**	**43.0**	**124**	**54.6**	**342**	**41.8**
6C	0		1	0.5	3	1.1	10	4.4	14	1.7
8	0		2	1.0	2	0.8	2	0.9	6	0.7
9N	0		1	0.5	1	0.4	1	0.4	3	0.4
10	0		7	3.3	4	1.5	4	1.8	15	1.8
11	1	0.9	9	4.3	9	3.4	8	3.5	27	3.3
12	0		0		1	0.4	3	1.3	4	0.5
15A	0		0		3	1.1	3	1.3	6	0.7
15B	0		4	1.9	15	5.7	6	2.6	25	3.1
16F	1	0.9	3	1.4	7	2.6	6	2.6	17	2.1
21	1	0.9	2	1.0	10	3.8	4	1.8	17	2.1
22F	1	0.9	4	1.9	6	2.3	3	1.3	14	1.7
23A	1	0.9	2	1.0	4	1.5	2	0.9	9	1.1
23B	0		2	1.0	3	1.1	3	1.3	8	1.0
31	0		0		1	0.4	5	2.2	6	0.7
33	1	0.9	3	1.4	3	1.1	4	1.8	11	1.3
35	4	3.4	0		3	1.1	7	3.1	14	1.7
38	0		0		1	0.4	0		1	0.1
Other^4^	0		9	4.3	9	3.4	6	2.6	24	2.9
NT	3	2.6	2	1.0	4	1.5	0		9	1.1
**Non PCV13**	**13**	**11.1**	**51**	**24.4**	**89**	**33.6**	**77**	**33.9**	**230**	**28.1**
Total episodes	117		209		265		227		818	

1 PCV7: number of isolates of serotypes included in the PCV7: serotypes 4, 6B, 9V, 14, 18C, 19F and 23F.

2 PCV10: number of isolates of serotypes 1, 5, and 7F, included in the PCV10 but not in the PCV7.

3 PCV13: number of isolates of serotypes 3, 6A, and 19A, included in the PCV13 but not in the PCV10.

4 Non-PCV13 serotype (2, 7A, 13, 17F, 18F, 19B, 24, 25, 28, 29, 34, 36, 39,41,47) who total number was less than 4 were grouped as “Others”.

Diversity increased from the pre-vaccine period to the 3 post-vaccine periods: 0.642 (95%CI 0.507–0.776) in the pre-vaccine period (1999–2001) to 0.844 (95%CI 0.782–0.907) in the second, 0.899 (95%CI 0.864–0.933) in the third period, and 0.844 (95%CI 0.774–0.915) in 2008–2010. When the PCV7 serotypes were excluded the significant difference between the pre-vaccine and the post-vaccine periods remained: 0.524 (95%CI 0.360–0.687), 0.814 (95%CI 0.733–0.894), 0.885 (95%CI 0.835–0.935), and 0.876 (95%CI 0.813–0.938) for the 1999–2001, 2002–2004, 2005–2007 and 2008–2010 periods, respectively.

### Mixed Infections

Among the 818 isolates, in 556 (68.0%), *S. pneumoniae* was isolated alone, in 214 (26.2%) with *H. influenzae* and in 48 (5.9%) with microorganisms other than *H. influenzae*: *S. aureus* (n = 23), *S. pyogenes* (n = 9), *M. catarrhalis* (n = 6), etc. Triple mixed infections were found in 12 episodes (1.5%), most of them (n = 7) with *S. aureus.*

When we divided the study in four 3-year periods, we observed 117 episodes in 108 children in 1999–2001, 209 episodes in 187 children in 2002–2004, 265 episodes in 215 children in 2005–2007 and 227 episodes in 198 children in 2008–2010. Thirty children had two or more AOM episodes in different periods.

By periods, the percentages of episodes with mixed infections were similar in the four study periods: 31.6% (n = 37/117) in the pre-PCV7 period and 31.6% (66/209), 32.8% (87/265) and 31.7% (72/227) for the early, intermediate and late post-PCV7 periods. The percentages of pneumococci and *H. influenzae* mixed infections showed no differences (23.1%, 28.2%, 29.1% and 22.5% for 1999–2001, 2002–2004, 2005–2007 and 2008–2010, respectively). No *H. influenzae* group b was found.

By age, the frequency of episodes of overall mixed infections or mixed infections with *H. influenzae* was similar in children under or over 2 years old: 175/565 (31.0%) overall and 140/565 (24.8%) with *H. influenzae* versus 87/253 (34.4%) overall and 74/253 (29.2%) with *H. influenzae* (p = 0.3 and p = 0.2 for overall and for *H. influenzae* mixed infections respectively). By gender, the frequency remained non-significant: 133 overall mixed infections and 111 with *H. influenzae* in 453 episodes in boys and 129 (p = 0.07) and 103 (p = 0.2) in 365 episodes in girls, respectively.

Together or individually, PCV7 serotypes were similarly found as single etiology or as mixed infections (total 125/556 and 68/262, respectively, p = 0.3). Serotypes 1 (20/556 versus 1/262, p = 0.004), 3 (75/556 versus 17/262, p = 0.003) and 19A (170/556 versus 57/262, p = 0.01) were found mainly as a single etiology. In contrast, serotype 15B was more frequently found in mixed cultures (13/262 versus 12/556, p = 0.03). No differences were found among others serotypes.

### Genotyping

Overall, 31 different PFGE patterns were identified among the random selection of 115 serotype 19A isolates characterized by PFGE (11, 27, 34, and 43 isolates per each study period, respectively). The high heterogeneity found within serotype 19A isolates was found in the four study periods with changes in the prevalence of specific clones throughout the study period. ST276 was the major clone circulating from 1999–2001 (3/11 isolates), 2002–2004 (9/27 isolates) and 2005–2007 (5/34 isolates). During the last period, 2008–2010, ST320 (19/43 studied isolates) was slightly more frequent than ST276 (15/43 isolates) which was the second most common genotype. ST320 was detected for the first time in this series in 2005. ST193 and ST199 clones were found in the four study periods, but minority clones such as ST62, ST63, ST202, ST220, ST994 and ST1201were only occasionally present. When all multidrug-resistant serotype 19A isolates (whether included or not in the random selection) were grouped, ST276 was the most prevalent clone (n = 69), followed by ST320 (n = 29). ST276 showed penicillin, erythromycin, tetracycline, and trimethoprim-sulfamethoxazole (SXT) MICs of 1–2, >4, >4 and 1–2 µg/mL, respectively. All ST320 isolates showed multiresistance with penicillin, erythromycin and SXT resistance and showed amoxicillin MICs ≥2 µg/ml (25/29, 88.2%, amoxicillin MICs were ≥4 µg/ml).

The 46 serotype 3 isolates characterized by PFGE showed three different patterns, corresponding to ST180, ST260 and ST1220. The most prevalent clone was ST180 (61.4%) and the least prevalent clone was ST1220 (4.5%), which belonged to the same clonal complex as ST260 (34.1%). ST180 and ST260 isolates were homogeneously distributed in the different periods. All serotype 3 isolates were penicillin- and erythromycin-susceptible (MICs <0.12 µg/ml and <0.5 µg/ml, respectively).

### Recurrences

#### Relapses

Relapses occurred in 21 children (Table 3). Fourteen children relapsed with an interval of less than 3 weeks, six between 21 and 30 days and one relapsed after 43 days. Risk factors for relapses were detected in only three children: adenoids and obstructive sleep apnea, atopy, Down syndrome and obstructive sleep apnea.

**Table 3:** Relapses. Patients with a second episode or more of infection in an interval of between 7 and 90 days caused by isolates with the same serotype and genotype.

Patient	Age	Gender	Date (year/month/day)	Isolation interval (in days)	PCV7	Serotype	MLST	Resistance pattern^1^
RL_1	1 year	Male	1999/01/19	43	None	19F	ST63	PEN,ERY,CLI,TET,SXT
			1999/03/08					
RL_2	2 years	Male	1999/04/30	7	None	14	ST156	PEN,SXT
			1999/05/07					
RL_3	1 year	Male	2000/03/01	14	None	14	ST1964	PEN,ERY,CLI,TET,SXT,CHL
			2000/03/15					
RL_4	1 year	Male	2000/05/25	27	None	14	ST156	PEN,SXT
			2000/6/21					
RL _5	8 months	Male	2001/11/23	19	None	6B	ST1624	PEN,ERY,CLI,TET,SXT
			2001/12/12					
RL _6	2 years	Male	2002/01/22	10	None	11	ST42	**–**
			2002/02/01					
RL _7	<1 month	Male	2002/12/17	20	None	19F	ST88	PEN, TET, SXT,CHL
			2003/01/06					
RL _8	7 months	Female	2003/05/02	17	None	23F	ST81	PEN,ERY,CLI,TET,SXT,CHL
			2003/05/19					
RL _9	10 months	Male	2003/05/19	11	None	19A	ST199	–
			2003/05/30					
RL _10	2 months	Female	2003/12/12	18	None	19F	ST63	PEN,ERY,CLI,TET
			2003/12/30					
RL _11	8 months	Male	2004/03/30	13	None	14	Slv1964	PEN,ERY,CLI,SXT
			2004/04/12					
RL _12	1 year	Female	2004/08/03	21	None	19F	ST424	–
			2004/08/24					
RL _13	6 months	Male	2007/04/19	27	Yes	19A	ST320	PEN,ERY,CLI,TET,SXT
			2007/05/16	23				
			2007/06/08	29				
			2007/07/07					
RL _14	7 months	Female	2007/06/07	25	Yes	19A	ST193	ERY,CLI,TET
			2007/07/02					
RL _15	4 months	Male	2008/10/11	10	None	19A	ST320	PEN,ERY,CLI,TET,SXT
			2008/10/21					
RL _16	1 year	Male	2009/05/15	9	Yes	19A	ST320	PEN,ERY,CLI,TET,SXT
			2009/05/24					
RL _17	7 months	Male	2009/11/19	30	Yes	19A	ST320	PEN,ERY,CLI,TET,SXT
			2009/12/19					
RL _18	5 months	Male	2009/11/24	18	Yes	19A	ST320	PEN,ERY,CLI,TET,SXT
			2009/12/12					
RL _19	11 months	Female	2009/12/10	12	None	19A	ST276	PEN,ERY,CLI,TET,SXT
			2009/12/22					
RL _20	6 months	Male	2010/02/16	30	Yes	19A	ST193	ERY,CLI,TET
			2010/03/18					
RL _21	11 months	Male	2010/05/26	7	Yes	19A	ST276	PEN,ERY,CLI,TET
			2010/06/02					

1 PEN = penicillin, ERY = erythromycin, CLI = clindamycin, TET = tetracycline, SXT = trimethoprim-sulfamethoxazole, CHL = chloramphenicol.

In the first two study periods (1999–2001 and 2002–2004), relapses were produced mainly by PCV7 serotypes and the percentage of relapses progressively decreased: there were five relapses among 108 children (4.6%) in the pre-PCV7 period, decreasing to 7/187 (3.7%) and 2/215 (0.9%) in the early and intermediate post-PCV7 periods (p for trend p = 0.04). In the late post-PCV7 period, relapses increased, with seven relapses among 198 (3.5%) children, all of them caused by serotype 19A isolates. Only seven relapsing children had previously received the PCV7 and all of them were infected with serotype 19A isolates. One of these vaccinated children relapsed four times at intervals of between 23 and 30 days with the multiresistant ST320 clone. Five months after the last relapse, this child had a fifth episode with the same clone, which was classified as a reinfection, based on the definition described in Material and Methods. No modification in the susceptibility pattern of relapsing isolates was observed in any of the children. Diminished susceptibility to penicillin (MIC >0.06 µg/mL) was observed in 76.2% of isolates from relapsing children, and amoxicillin resistance (MIC >2 µg/mL) in 33.3% (n = 7). Isolates from another four children showed an amoxicillin MIC of 1–2 µg/mL (52.4% of relapsing children had isolates with an amoxicillin MIC >0.5 µg/mL). Moreover, erythromycin resistance was observed in isolates from 71.4% of relapsing children.

#### Reinfections

There were 86 children (48.8% boys) with at least two episodes of infection: 68 children had one episode of reinfection, 13 had two, four had three, and one child had four episodes, making a total 110 episodes of reinfection (196 episodes of infection). PCV7 serotypes produced 26 episodes of reinfection in 24 children, of which only 8 children (9 episodes) were vaccinated at the time of the reinfection episode. Reinfections were more common in children under 24 months than in older children. Thirty infants younger than 1 year old (30/229, 13.1%) and 39 one-year-old children (39/270, 14.4%) acquired a second episode of infection versus 10 children aged 2 years old (10/105, 9.5%), five children aged 3 years old (5/63, 7.9%) and only one child aged 4 years old (1/41, 2.4%).

Reinfections were caused by an isolate with a different serotype than that causing the first infection in 69 children and by an isolate of the same serotype (RI-SS) in 17 children. Of these 17 children with RI-SS (Table 4) only three (17.6%) were unvaccinated and two of the reinfections were caused by serotype 19F. Serotype 19A was the most frequent serotype causing RI-SS (n = 9, 52.9%) while serotypes 3, 6C, 15A, 15B, 22F and 23A, caused one each.

**Table 4:** Patients with reinfections caused by the same serotype as the initial infection.

Patient	Age	Gender	Date (year/month/day)	Isolation interval (in days)	PCV7^a^	Serotype	PFGE pattern b	MLST
RI_1	1 year	Male	1999/12/11	128	None	19F	K	ST202
			2000/04/17		None	19F	B	ST276
RI _2	8 months	Female	2004/03/02	154	None	19F	E	ST424
			2004/08/03		None	19F	E	ST424
RI _3	1 year	Male	2005/02/07	746	Yes	3	D	ST180
			2007/02/23		Yes	3	D	ST180
RI _4	5 months	Female	2006/12/09	444	Yes	19A	C	ST199
			2008/02/26		Yes	19A	B	ST276
RI _5	10 months	Male	2007/02/05	1199	Yes	15A	O	ST2613
			2010/05/19		Yes	15A	P	NEW SLV193
RI _6	7 months	Male	2007/03/01	692	Yes	22F	H	ST30
			2009/01/21		Yes	22F	Q	ST433
RI _7	6 months	Male	2007/04/19	236	Yes	19A	A	ST320
			2007/12/11		Yes	19A	A	ST320
RI _8	1 year	Male	2007/06/03	145	Yes	19A	M	ST994
			2007/10/26		Yes	19A	C	ST199
RI _9	7 months	Female	2007/06/07	45	Yes	19A	J	ST193
			2007/07/22		Yes	19A	B	ST276
RI _10	1 year	Female	2008/01/23	365	Yes	23A	C	ST2829
			2009/01/22		Yes	23A	C	ST2829
RI _11	9 months	Female	2008/05/21	118	Yes	19A	B	ST276
			2008/09/16		Yes	19A	A	ST320
RI _12	1 year	Female	2008/07/08	400	Yes	15B	R	NEW SLV5216
			2009/08/12		Yes	15B	R	NEW SLV5216
RI _13	2 years	Female	2008/10/14	213	Yes	6C	N	ST1692
			2009/05/15		Yes	6C	L	ST386
RI _14	1 year	Male	2009/06/10	167	Yes	19A	A	ST320
			2009/11/24		Yes	19A	A	ST320
RI _15	1 year	Male	2009/10/13	267	Yes	19A	I	ST62
			2010/07/07		Yes	19A	C	ST199
RI _16	1year	Female	2009/12/10	111	Yes	19A	A	ST320
			2010/03/31		Yes	19A	A	ST320
RI_17	4 years	Male	2010/04/06	18	None	19A	C	ST199
			2010/04/24		None	19A	B	ST276

a PCV7∶7-valent pneumococcal conjugate vaccination (PCV7 included serotypes: 4, 6B, 9V, 14, 18C, 19F and 23F).

b Pulse-field gel electrophoresis pattern, arbitrary named MLST = multilocus sequence typing.

After PFGE and MLST analysis, we observed that 10 (58.8%) of the 17 RI-SS had a different genotype, giving a total of 79 children with reinfections caused by a different strain and 7 with reinfections caused by isolates with the same genotype but with an interval of more than 90 days between the first episode and the reinfection. Among these 7 children with reinfections caused by the same genotype (same strain), three reinfections were caused by serotype 19A, all of which were ST320. None of these children showed anatomical lesions, chromosome anomalies, immunodeficiency, or any other apparent risk factor.

By age, no differences were found in the frequency of RI-SS when we grouped children into those aged younger or older than 2 years old (15/499 *versus* 2/209; p = 0.2) and into those younger than 1 year (7/229), those aged 1 to 3 years (8/438), and those aged more than 3 years (1/41) (p = 0.6).

## Discussion

AOM is probably the most common infectious disease in young children treated by physicians and produces frequent recurrences [13]. Compared with other bacteria causing AOM, *S. pneumoniae* otitis has been associated with more severe signs and symptoms and more complications [14].

In this study, we analyzed the dynamics of *S. pneumoniae* clones causing AOM between 1999 and 2010 in a region of northern Spain and their involvement in recurrences. Despite the large number of studies of invasive pneumococcal disease (IPD) in the last few years, there is little information on pneumococcal AOM with full characterization of isolates from a single region over a broad time period. All capsulated isolates were serotyped, and only 9 of 818 isolates were unencapsulated, the uniqueness of this finding being well known [15].

One limitation of this study is that the number of isolates included in each period depended on physician demand. Considering that many patients with mild AOM never attend their physician and spontaneously cure, we could not determine changes or real incidence of AOM in our region. Unlike the diagnostic and therapeutic procedures performed in other regions, the tympanic membrane puncture was an infrequent practice in our region, for this reason all episodes in the present study were diagnosed after spontaneous middle-ear drainage, although sampling was not always performed when the perforation of the ear-drum was visually evident.

AOM is most prevalent in children under 2 years of age, with a peak incidence in children aged between 6 and 18 months [16]. In the present series, 69.1% of episodes occurred before the second year of age (70.5% of children had their first episode at this young age and 54.9% of them were boys).

Mixed infections were common and the most frequent accompanying bacterium was non-encapsulated *H. influenzae*. The association of these two otopathogens in AOM has been largely demonstrated [17], [18]. Several studies suggested the possibility of an increase of *H. influenzae* AOM after the introduction of PCV7 [19]. In our experience, the number of AOM caused by *H. influenza*e increased (data not shown) but mixed infections with pneumococcus and *H. influenzae* were similar in the four study periods. Mixed infections were also similar in children under and over 2 years old. Serotypes 1, 3 and 19A were mainly found as single etiology infection, which could suggest a higher invasive disease potential.

As the number of colonies selected for serotyping as well as the methodology used may influence the number or diversity of serotypes found in the samples, in the present work we selected 3 or 4 isolated colonies from the primary culture plate to perform the initial serotype characterization. The frequency of the different serotypes causing pneumococcal diseases depends mainly on two factors: the capacity to spread and the disease potential for each site of infection [20]. As pneumococcal diseases start with nasopharynx colonization, a reduction in the number of infections caused by vaccine serotypes could be expected, since PCV7 has been demonstrated to reduce vaccine serotype carriage, decreasing the circulation of these serotypes [21]. It is well known that the introduction of the PCV7 has been accompanied by changes in the distribution of serotypes causing AOM [2], [5], [6]. In the present study, we observed a dramatic reduction in the proportion of PCV7 serotypes causing AOM, from 62.4% in the pre-vaccine period to 2.2% in 2008–2010. A similar reduction was observed in another Spanish study: from 70.7% in 1997–2000 to 10.6% in 2009 [22].

Among PCV7 serotypes, 19F was the most common, being especially prevalent in the first two periods. This association of 19F with AOM has also been observed in other studies [20], [23]. The initial data suggested that the protection of PCV7 against serotype 19F infections, especially AOM, was lower than that against other serotypes included in the PCV7 [6], [24]. However, we found that 19F was the PCV7 serotype with the largest drop in percentage across the four study periods (from 20.5% in the pre-PCV7 period to 1.3% in the last period), a decrease that seems not to be consistent with a questionable protective effect against serotype 19F of the PCV7. Serotype 19F was also common in the present work among unvaccinated children with recurrences.

There is currently some concern about the finding of discrepant results in the serotyping obtained by Quellung and PCR capsular typing in a few serotype 19F and 19A isolates [25]; however, in our series, serological and PCR results agreed for all 19F and 19A isolates.

The reduction in PCV7 serotypes causing AOM was accompanied by an increase in the rate of non-vaccine serotypes, especially of serotypes 19A and 3 and, to a lesser extent, of other less frequent serotypes or serogroups such as 6C, 15B, 11, 16F and 21. Serotype 6C was the third most frequent serotype from 2008–2010, a serotype recently described and detected for the first time in 2002 in the present study, although it was not detected in any IPD in children in our region until 2012 [26].

Serotype 19A was the most prevalent serotype in the three post-PCV7 periods and showed a similar prevalence in the pre-PCV7 period to serotype 19F, the most prevalent PCV7 serotype. The increased presence of serotypes 19A and 3 in AOM has also been found in other works [6], [20], [22]. In a study of IPD in children in our region in the same years, we found a decrease of infections due to PCV7 serotypes and an increase of serotype 19A similar to those observed for AOM [27]. However, there was no increase in the incidence of serotype 3 invasive infections in children, confirming the stronger association of serotype 3 with respiratory infections, both in the lower respiratory tract of older adults [28], [29] and AOM in children [6], [22], [30]. Genotyping analysis of serotypes 19A and 3 isolates also showed a different distribution: while serotype 3 isolates belonged to only three well-known clones (ST180, ST260, and ST1220), serotype 19A isolates showed greater heterogeneity.

Phenotypic and genotypic analysis showed major changes in the dynamics of infection over the 12-year period. Genetic studies identified several apparently identical isolates as different strains. Two isolates of the same serotype, consecutively isolated from the same children, were frequently found to have different genotypes. Indeed, among 19 episodes of reinfection with the same serotype, the same strain (same genotype) was only found in 9.

Among serotype 19A isolates, ST276 was the most prevalent throughout the study. Furthermore, ST276 was the major clone circulating in the periods 1999–2001, 2002–2004 and 2005–2007, and was second only to the predominant ST320 from 2008–2010. In 2004–2005, we performed a study of carriers in healthy children attending a daycare center in this same region and observed that 19A was also the most prevalent serotype [31]. However, in that study of carriers, the most prevalent genotypes of serotype 19A were ST193 and ST199, and no ST276 or ST320 isolates were found in the sample studied [31]. In Asia and the USA, ST320 has been described as a highly prevalent clone showing a worldwide distribution and rapid and broad spread among children. To date, ST320 has had limited diffusion among the adult Spanish population [32].

The diversity of a community depends on the richness (number of species) and relative abundance of each species. The Simpson diversity index, used in this study to analyze the serotype diversity in distinct periods, takes into account the number of serotypes present, as well as the abundance of each serotype. In the present work, the serotype diversity increased from the first period (pre-vaccine) to the following post-vaccine periods. The increase was not only a consequence of the removal of competitive PCV7 types because excluding PCV7 serotypes, the difference between the first and remaining periods persisted. The fall in the diversity in the fourth period was not significant despite the disproportionate presence of serotype 19A isolates. Antimicrobial resistance decreased from 1999–2001 to 2005–2007, but in 2008–2010, resistance rates again increased due to the spread of multiresistant serotype 19A isolates [33].

Recurrent AOM is associated with several factors, including early onset of the disease [34]. Relapses in the present work occurred mainly among male infants that were unvaccinated or infected by isolates of serotypes not included in the PCV7 and were very often caused by antimicrobial resistant isolates. In the first 6 years (1999–2004) of the study, more than 80% of relapses were caused by serotypes included in the PCV7, while in the second 6-year period (2005–2010) 100% were caused by serotype 19A. We did not find any relapses caused by a PCV7 serotype in vaccinated children, once again, underscoring the protective effect of the vaccine. Reinfections or new episodes of AOM caused by a different strain from the original infection or by the same strain but after a long period of time were detected in 12.1% of children and 13.4% of episodes. Episodes of reinfections were more frequent in younger children. The number of episodes of reinfection in both genders was similar (48.8% in boys). If reinfection was due to the persistence of factors predisposing to infection, RI-SS could indicate a lack of adaptive immunological response. Serotype 19A caused 9/17 (52.9%) RI-SS, a ratio higher than that corresponding to the representation of serotype 19A in the whole sample (p = 0.03). Apparently, these children had no predisposing factors for these RI-SS, although a specific immunological study of these children was not performed. A less robust adaptive immune response to several *S. pneumoniae* proteins has been observed in children with relapses and reinfections [35]. Because the immune response depends not only on the host but also on the antigen, we speculate that 19A serotype isolates or any of its clones may have been the cause of a less adaptive immune response of the host. All together, the high number of total AOM cases caused by serotype 19A and the high percentage of relapses and RI-SS found in the present study seem to suggest an immunological hyporesponsiveness to previous colonization or infection with serotype 19A isolates that should be taken into account in future studies. The high burden of serotype 19A in AOM recurrences has also been previously reported [36].

In the present study, the criterion for considering a recurrence caused by the same strain as a relapse or reinfection was arbitrarily chosen because the length of time a pneumococcus stays in the middle ear is unknown. Some episodes of reinfection could probably have been a very late relapse, as occurred with relapsing child number 13, who had four recurrences with a serotype 19A ST320 between the ages of 6 and 14 months.

The two most prevalent serotypes causing AOM in the last time period in the present and in other similar studies were serotypes 19A and 3. Since both serotypes are included in the new PCV13, it will be interesting to measure the impact of PCV13 in the epidemiology of these two as well as in that of the other serotypes included in the vaccine. Assuming that in the next few years a drop in AOM caused by serotypes 19A and 3 might occur, continued surveillance is required because, in the absence of the competitive effect of these prevalent serotypes, other serotypes not present or poorly represented in the pre-vaccination period such as serotypes 6C, 15B, 11, 16F or 21 could become predominant.
